# Rapid and Non-Destructive Prediction of Moisture Content in Maize Seeds Using Hyperspectral Imaging

**DOI:** 10.3390/s24061855

**Published:** 2024-03-14

**Authors:** Hang Xue, Xiping Xu, Yang Yang, Dongmei Hu, Guocheng Niu

**Affiliations:** 1 College of Optoelectronic Engineering, Changchun University of Science and Technology, Changchun 130022, China; xuehang@beihua.edu.cn (H.X.); yy18855187372@163.com (Y.Y.); 2 College of Electronic and Information Engineering, Beihua University, Jilin 132021, China; hudongmei@beihua.edu.cn (D.H.); niuguochengjilin@163.com (G.N.)

**Keywords:** hyperspectral imaging, moisture content, maize seed, non-destructive, visualization

## Abstract

The moisture content of corn seeds is a crucial indicator for evaluating seed quality and is also a fundamental aspect of grain testing. In this experiment, 80 corn samples of various varieties were selected and their moisture content was determined using the direct drying method. The hyperspectral imaging system was employed to capture the spectral images of corn seeds within the wavelength range of 1100–2498 nm. By utilizing seven preprocessing techniques, including moving average, S–G smoothing, baseline, normalization, SNV, MSC, and detrending, we preprocessed the spectral data and then established a PLSR model for comparison. The results show that the model established using the normalization preprocessing method has the best prediction performance. To remove spectral redundancy and simplify the prediction model, we utilized SPA, CASR, and UVE algorithms to extract feature wavelengths. Based on three algorithms (PLSR, PCR, and SVM), we constructed 12 predictive models. Upon evaluating these models, it was determined that the normalization-SPA-PLSR algorithm produced the most accurate prediction. This model boasts high $R_{C}^{2}$ and $R_{P}^{2}$ values of 0.9917 and 0.9914, respectively, along with low RMSEP and RMSECV values of 0.0343 and 0.0257, respectively, indicating its exceptional stability and predictive capabilities. This suggests that the model can precisely estimate the moisture content of maize seeds. The results showed that hyperspectral imaging technology provides technical support for rapid and non-destructive prediction of corn seed moisture content and new methods in seed quality evaluation.

## 1. Introduction

Maize is an important grain crop and cash crop in China. It is very important to control the moisture content in the process of storage and breeding. After threshing, the embryo structure of maize is exposed in the external environment, which makes maize seeds vulnerable to the interference of that environment, resulting in low storage stability. During storage, it is of great use to keep the moisture content of corn grain below 13% in order to reduce the metabolic rate of corn grain in the sink, prevent excessive heat generation from causing mildew, and ensure the nutritional content and seed vigor of seeds [1,2,3]. In addition, in the process of breeding, the maize seeds stored in the storehouse for a long time have very high requirements for the temperature and humidity of the environment, and the moisture content carried by the seeds when they are stored in the storehouse has an important impact on the germination rate of the seeds in the future [4]. Therefore, the control and detection of moisture content is the key link to ensure the quality of corn seeds during the process of corn warehousing.

At present, the moisture content of maize seeds is usually detected by drying or chemical methods to remove the water in the corn grains, after which the moisture content of the sample can be calculated [5,6]. Although these methods have high detection accuracy, they destroy the activity of the seeds. If there are more batches of corn, more samples need to be taken, which consumes time and labor.

Hyperspectral imaging (HSI) integrates the advantages of spectroscopy and imaging, enabling simultaneous non-destructive testing of multiple targets and visualization of material composition content [7]. This technology has the characteristics of multiple continuous wavebands, high spectral resolution, and “map one”, meeting the demands of rapid non-destructive testing. In recent years, it has been studied widely and in depth, and applied in the quality detection of agricultural products and food [8,9,10,11,12,13,14,15,16,17]. Nicola et al. used HSI to detect the moisture and lipid content of single coffee bean and visualize their distribution [18]. Xu et al. collected hyperspectral images of single cucumber seeds in the range of 400–1000 nm and 1050–2500 nm, and then predicted the moisture content of single cucumber seeds based on the two bands and conducted visualization analysis. It was found that the predicted effect of moisture content was greater in the range of 1050–2500 nm [19]. Jennifer et al. performed moisture content detection and visualization of single peanut kernels in the range of 900–1700 nm, but only used the weighted regression coefficient method to extract characteristic wavelengths [20]. 

Wakholi et al. used HSI to measure the vitality of corn seeds and visualized the results [21]. Zhang et al. combined HSI and a deep convolutional generative adversarial network to predict the oil content of a single maize kernel, the results of which indicated the potential of HSI in the oil detection of maize seeds [22]. As for moisture content detection in maize seeds, some scholars have carried out research using his; for example, Lian et al. combined HSI and RF algorithms to measure the moisture content of fresh-eating fruit corn, with an accuracy rate of 82.5% [23]. This indicates the feasibility of HSI-based corn moisture detection, although the precision was not high, as no in-depth study was conducted on the effectiveness of different algorithms. Wang et al. established a CARS-SPA-LS-SVM model to measure the moisture content of seeds; the accuracy of this model reaches 93.11% [24]. However, the study used a single type of sample with a wide range of water contents by artificially increasing the moisture levels of the seeds, which restricted the model’s applicability. 

In conclusion, HSI is feasible for rapid detection of moisture content in maize grains, and 1000–2500 nm is the ideal wavelength for moisture content detection. However, the study of corn seed in Northeast China is not sufficient, as it has included no research on spectral preprocessing and selection methods for characteristic wavelengths, and has not obtained high accuracy. In this study, we selected 80 maize varieties as the research object, providing a diverse data set that facilitates the evaluation of the measurement accuracy and reliability of hyperspectral imaging technology under different genetic backgrounds. We compared seven preprocessing methods and three feature wavelength selection methods to find the optimal prediction model. At the same time, a visualization study was conducted on the water content of corn seeds to enhance the practicality and scalability of the technology. Through this study, we can provide an experimental basis for the application of HSI in the quality detection of seeds and provide technical support for moisture content detection in the process of maize harvesting, storage, and processing.

## 2. Materials and Methods

### 2.1. Samples

**Figure 1:**
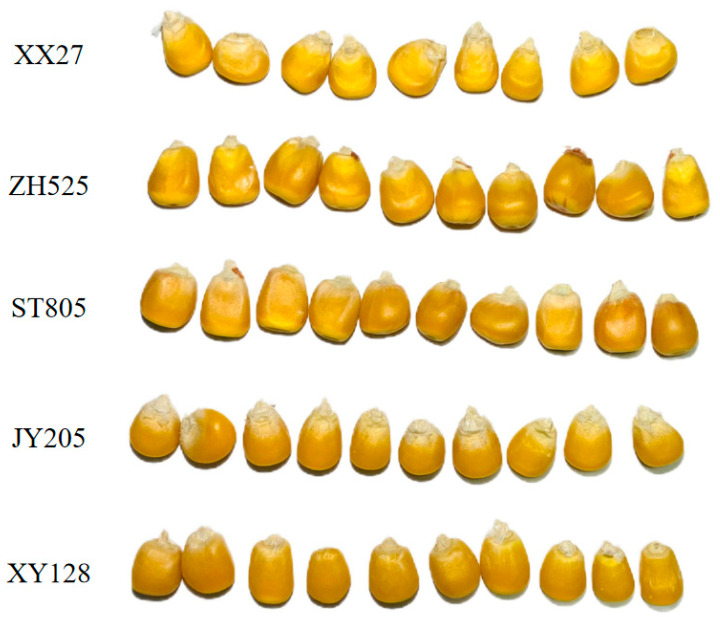
Five kinds of seeds in the experimental samples.

The maize seeds used in the experiment were provided by Jilin Guangde Agricultural Technology Co., Ltd., Tonghua, Jilin, China (located at 42°39′ N and 126°08′ E), including 80 varieties such as XX27, ZH525, ST8, JY2, XY128, etc. These samples were different types of hybrid seeds obtained in the same growth environment in the same year, all seeds were uncoated, and there was no significant difference in surface properties. Figure 1 depicts five kinds of seeds in the experimental samples. 

A 100 g sample of each variety was placed in a petri dish and allowed to stand in the laboratory for 72 h to stabilize the internal moisture distribution of the seeds. We then collected hyperspectral images of the samples and measured the moisture content of each variety of corn sample using the direct drying method described in the GB5009.3-2016 National Food Safety Standard—Determination of Moisture in Food [25]. We measured the samples three times for each variety and took the average as the moisture content of that variety of corn seeds.

### 2.2. Experimental Equipment

The experiment utilized a hyperspectral imaging system to collect spectral images of various corn varieties. The system includes a 150 W halogen lamp symmetrical linear light source (IT3900, Illumination Technologies Inc., Liverpool, NY, USA), a 1000–2500 nm spectral module (ImSpector N25E, Spectral Imaging Ltd., Oulu, Finland), a resolution 1600 × 1200 area array CCD camera (ICL-B1410, IMPERX Inc., Boca Raton, FL, USA), a precision mobile control platform (IRCP-0076-400, Isuzu Optics Corp., Taiwan, China), and a dark box for minimizing environmental interference (1.2 × 1.4 × 0.5 m), as well as a computer for control and data acquisition. Image acquisition and displacement control were managed by spectral processing software (Spectral Image-N25E, Isuzu Optics Corp., Taiwan, China), while data processing and model establishment were carried out using Matlab.

Before image acquisition, we adjusted the object distance, exposure time, focal length, and moving speed of the optical system to ensure that the captured image shape was clear and accurate. After multiple experiments, the instrument parameters during the acquisition process were set as follows: the acquisition range of the hyperspectral imaging system was 935.5–2539 nm, the spectral resolution was 6.3 nm, the number of bands collected was 256, the lens focal length was 36 cm, the exposure time was 10 ms, and the moving speed of the platform was 7 mm/s.

During image acquisition, black and white noise is acquired for black and white correction to reduce or eliminate the effects of dark current, stray light, and noise interference from charge-coupled devices in hyperspectral cameras [26,27]. The correction formula is:

(1) $$R=\frac{I_{raw}-I_{dark}}{I_{white}-I_{dark}}$$

where *R* is the corrected image, *I_raw_* is the original image, *I_white_* is the fully white-calibrated image, and *I_dark_* is the fully black-calibrated image.

### 2.3. Data Processing and Modeling Methods

#### 2.3.1. Preprocessing Methods

When imaging a hyperspectral imaging system, the data are frequently affected by factors such as the instrument background, uneven particle distribution, or different particle sizes, as well as instrument signal noise. To enhance the model’s prediction accuracy and stability, the collected data need to be preprocessed to remove interference factors. Preprocessing methods can be categorized into four types: scatter correction, baseline correction, smoothing, and scaling [28,29,30]. Due to the variability of instrumental errors and environmental factors, there is currently no universal and highly versatile spectral preprocessing algorithm, nor is there a widely recognized evaluation parameter.

The preprocessing methods used in this article include: moving average, S–G smoothing, baseline, normalization, standard normal variate (SNV), multivariate scatter correction (MSC), and detrending. A PLSR model was developed for the preprocessed spectral data to determine the optimal preprocessing method.

#### 2.3.2. Successive Projections Algorithm (SPA) Method

SPA is a forward variable dimensionality reduction algorithm proposed by Araujo et al., that minimizes collinearity in vector space. It can eliminate redundant information in the original spectral data, and thus facilitate spectral feature wavelength selection [31,32]. SPA is a forward selection method, which starts with one wavelength and merges a new wavelength at each iteration until all wavelengths are merged. The goal is to solve the collinearity problem and select wavelengths with minimal redundancy in information content [33]. The specific implementation steps of SPA are as follows:

1. Set the number of selected variables as n, and choose any column ($x_{j}$) in the spectral matrix X as the initial wavelength. The position of $x_{j}$ in the spectral matrix is marked as g(0), hence $x_{j}$ can be represented as $x_{g}(0)$.
2. Denote the set of remaining column vector positions as k:
  (2) $$s=\left\{j,\;1\leq j\leq J,\;j\notin g(0),\;g(1),\cdots ,\;g(n-1)\right\}$$
  where J is the number of columns in the spectral matrix X.
3. Compute the projections of $x_{j}$ onto the remaining column vectors separately:
  (3) $$P_{x_{j}}=x_{j}-\left[x_{j}^{T}x_{g(n-1)}\right]x_{g(n-1)}{\left[x_{g(n-1)}^{T}x_{g(n-1)}\right]}^{-1},\;j\in k$$
4. Extract the spectral wavelength of the maximum projection vector, denoted as:
  (4) $$g\left(n\right)=arg\left(max\left(\left\|P_{x_{j}}\right\|\right)\right),\;j\in k$$
5. Take the maximum projection value $g\left(n\right)$ as the initial value for the next iteration, return to step two, and perform cyclic calculations.
6. The combination of all bands obtained by dimensional reduction is denoted as S:
  (5) $$S=\left\{X_{g(j)};\;j=0,\;1,\cdots ,\;n-1\right\}$$

#### 2.3.3. Competitive Adaptive Reweighted Sampling (CARS) Method

CARS is a feature selection method that combines Monte Carlo (MC) sampling with Partial Least Squares (PLS) model regression coefficients, mimicking the principle of “survival of the fittest” in Darwinian theory [34,35]. In the CARS algorithm, adaptive weighted sampling is used to retain points with larger absolute values of regression coefficients in the PLS model as a new subset, removing points with smaller weights, and then establishing a PLS model based on the new subset. After multiple calculations, the wavelengths in the subset with the minimum RMSECV for the PLS model are selected as feature wavelengths. The specific process of the CARS algorithm is as follows:

1. By employing the MC sampling method, a fixed number of samples is randomly selected each time from the calibration set for the modeling set, while the remaining samples form the prediction set for building the PLS model. The number of MC samples (N) must be predetermined.
2. The weight of the absolute value of the regression coefficient in the PLS model for each iteration is calculated, denoted as $w_{i}$:
  (6) $$w_{i}=\frac{\left|B_{i}\right|}{\sum_{i=1}^{m}\left|B_{i}\right|}$$
  where $B_{i}$ represents the regression coefficient for the ith variable, and m represents the number of variables remaining in each sample.
3. The wavelength with a minor $w_{i}$ is removed through the Exponential Decay Function (EDF). At the ith time when establishing a PLS model through MC sampling, the proportion of retained wavelength points based on EDF is $r_{i}$:
  (7) $$r_{i}=\mu e^{-ki}$$
  where n is the number of original wavelength points, μ and k are constants, $\mu ={\left(n/2\right)}^{1/\left(N-1\right)}$, and $k=\ln\left(n/2\right)/\left(N-1\right)$.
4. During each sampling, the number of wavelength variables selected for PLS modeling using adaptive weighted sampling (ARS) is $r_{i}\times n$, and the RMSECV is calculated.
5. After repeating N times of sampling, the CARS algorithm yields N sets of candidate feature wavelength subsets and their corresponding RMSECV values. The subset of wavelength variables corresponding to the minimum RMSECV value is chosen as the feature wavelengths.

#### 2.3.4. Uninformative Variable Elimination (UVE) Method

The UVE algorithm can remove wavelength variables with a small effect on modeling co-efficiency and select characteristic wavelength variables [36]. Its main idea is to introduce artificial random noise information and combine it with PLS to establish a regression cross-validation model. The quotient of the mean and standard deviation of the regression coefficients is calculated as an evaluation index to measure the importance of the characteristic wavelength variables. At the same time, when introducing random noise, the maximum value of the noise matrix is used as the upper and lower limits of the algorithm threshold. The characteristic variables with a result higher or lower than the threshold are selected as the final optimized feature vector information.

There are n samples, $X_{n\times p}$ is the independent variable matrix, $Y_{n\times 1}$ is the dependent variable vector, and the PLS model selects the optimal number of principal factors as k. The specific algorithm is analyzed as follows:

1. $G_{n\times p}$ is a random noise matrix. Combine X and G to form a matrix ${XG}_{n\times 2p}$, where the first p columns of the matrix are X and the last p columns are G.
  (8) $${XG}_{n\times 2p}=\left[X,G\right]$$
2. Establish a PLS regression model for ${XG}_{n\times 2p}$ and $Y_{n\times 1}$, and obtain the regression coefficient matrix B and its regression vector b.
3. The average value and standard deviation C of the regression vector b can be obtained through the regression coefficient matrix B. The calculation formula for C is as follows:
  (9) $$C_{i}=\frac{mean(b_{i})}{stb(b_{i})}$$
4. The threshold value of standard deviation C is $C_{max}=max\left(\left|C\right|\right)$. If $C>C_{max}$, then the variable is the preferred eigenvector, and the selected subset is the feature wavelength set extracted by the UVE algorithm.

#### 2.3.5. Model Building and Evaluation

Partial Least Squares Regression (PLSR), Principal Component Regression (PCR) and Support Vector Machine Regression (SVMR) were used to develop the quantitative spectral analysis model for the moisture content of maize seeds. The performance of the models was evaluated mainly by the coefficient of determination ($R^{2}$) and root mean square error (RMSE) [37,38].

The calculation formula for $R^{2}$ is:

(10) $$R^{2}=\frac{\sum_{i=1}^{N}\left(x_{i}-\bar{x}\right)\left(y_{i}-\bar{y}\right)}{\sqrt{\sum_{i=1}^{N}{\left(x_{i}-\bar{x}\right)}^{2}+\sum_{i=1}^{N}{\left(y_{i}-\bar{y}\right)}^{2}}}$$

where $x_{i}$ is the actual measured value, $y_{i}$ is the predicted value, $\bar{x}$ is the average measured value, and $\bar{y}$ represents the average predicted value. $R^{2}$ is the coefficient of determination with a value range of [0, 1]. The closer $R^{2}$ is to 1, the better the prediction effect of the regression model.

The calculation formula for RMSE is:

(11) $$RMSE=\sqrt{\frac{1}{n}\sum_{i=1}^{n}{\left(y_{i}-\hat{y_{i}}\right)}^{2}}\;$$

where n is the number of samples, $y_{i}$ is the actual value of the *i*th sample, and $\hat{y_{i}}$ is the predicted value of the *i*th sample.

During the modeling process, the closer the $R_{C}^{2}$ and RMSEC of the model are to 1 and 0, the better the fitting effect and stability of the model, and the better it captures data patterns with lower error. During prediction, the closer $R_{P}^{2}$ and RMSEP are to 1 and 0, the stronger the predictive ability of the model, which can accurately predict future data based on existing data. In model validation, the closer $R_{CV}^{2}$ and RMSECV are to 1 and 0, the better the model performs in cross-validation, indicating that the model has good generalization ability and can maintain stable performance on different data sets. If the values of $R_{C}^{2}$ and $R_{P}^{2}$ are large with minimal difference, and the values of RMSEC and RMSEP are small with minimal difference, the model’s consistent performance across various metrics indicates its high reliability and credibility.

## 3. Results and Discussion

### 3.1. Sample Division

The algorithm for sample set partitioning based on joint X–Y distance (SPXY) was used to divide the samples into a calibration set and a prediction set according to the ratio of 4:1. The moisture content of the samples is shown in Table 1. The range of moisture content for the calibration set samples covers the range of the prediction set, indicating that the sample set division is reasonable.

### 3.2. Spectral Curve Analysis

**Figure 2:**
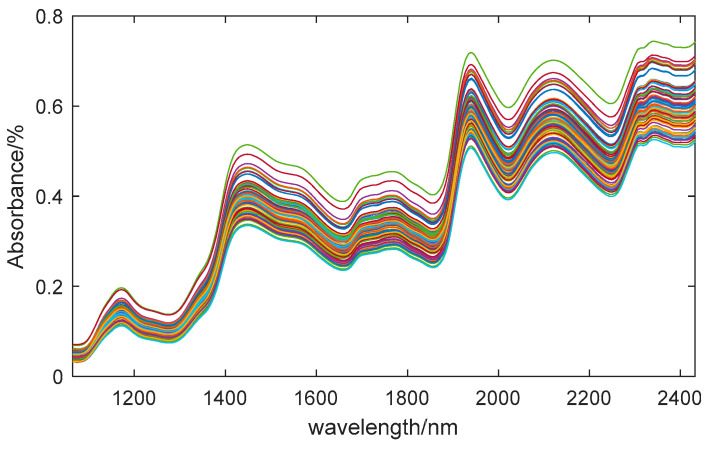
Reflectance curves of spectrum. (Different color curves represent different samples).

In the experiment, we obtained hyperspectral data with a wavelength range of 935.5–2539 nm, containing 256 bands. However, the initial and final sections were significantly affected by noise during the data acquisition. To ensure the accuracy of the research, we excluded these sections during analysis. Therefore, we used the middle 218 bands, which have a wavelength range of 1065–2432 nm, for in-depth exploration. The average spectral curve of 80 samples is shown in Figure 2. According to existing research, the absorption band of the O–H bond in water molecules in maize seeds is between 920 nm and 1950 nm [39]. As shown in the figure, the absorption peak at 1450 nm is related to the overtone vibration of the O–H bond, while the absorption peak at 1940 nm represents the combination frequency characteristic of the O–H bond [40]. These two peaks are characteristic bands of moisture content.

### 3.3. Spectral Preprocessing

In order to reduce the influence of irrelevant information and noise on spectral data, it is necessary to preprocess the spectral data. The spectral data were preprocessed using seven methods: moving average (window size of 7), S–G smoothing (window size of 7, polynomial order of 2), baseline, normalization, SNV, MSC, and detrending (polynomial order of 2). The PLSR model takes into account the relationship between independent and dependent variables, allowing for regression modeling under conditions of severe multicollinearity among independent variables. Therefore, the PLSR model was selected to compare the effects of different preprocessing methods. The leave-one-out cross-validation method was employed to calculate the root mean square error of cross-validation (RMSECV) as an evaluation metric for the model. After processing the spectral data, the PLSR models were built separately, and the preprocessing results are shown in Table 2. As shown in Table 2, the RMSECV for the prediction model without preprocessing is 0.0632, and the coefficient of determination ($R_{C}^{2}$) is 0.9772. After preprocessing, the stability of the model and the performance of cross-validation were enhanced. Specifically, the model processed by the normalization method exhibited the minimum RMSECV of 0.0410 and the highest $R_{C}^{2}$ of 0.9890. Therefore, this paper will be analyzed based on the data after normalization preprocessing.

### 3.4. Feature Wavelength Extraction

Hyperspectral images have huge spectral band resources, which lead to an increase in the correlation between adjacent band images and generate a large amount of redundant information, creating great difficulties for data analysis and modeling. Therefore, it is necessary to reduce the dimensionality of hyperspectral images through feature selection and extraction, and express the information of the overall data set with a small number of variables. In this study, SPA, CARS, and UVE were used to extract the feature wavelengths from the spectral data after pretreatment of maize seeds.

#### 3.4.1. Feature Wavelengths Extracted by SPA

**Figure 3:**
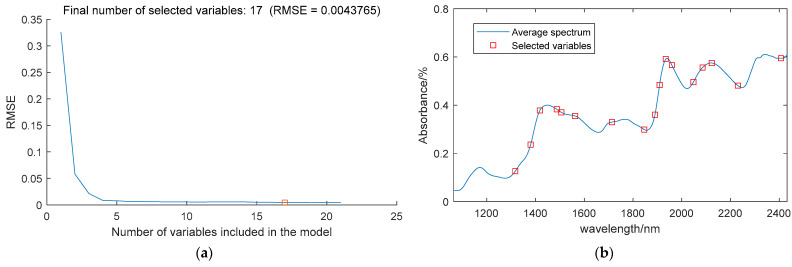
SPA feature extraction results of moisture content. (**a**) Correlation between RMSE and the number of variables. (**b**) Location of the characteristic wavelengths.

SPA was used to extract the characteristic wavelengths of the moisture content. Figure 3a illustrates the variation in RMSE as the number of variables increases; when the number of variables is 17, the minimum RMSE is 0.0044. Figure 3b illustrates the locations of the selected characteristic wavelengths. The extracted wavelengths include 1317 nm, 1380 nm, 1418 nm, 1487 nm, 1506 nm, 1562 nm, 1714 nm, 1846 nm, 1890 nm, 1909 nm, 1934 nm, 1959 nm, 2048 nm, 2085 nm, 2123 nm, 2230 nm, and 2407 nm, making up 7.8% of the entire spectral range.

#### 3.4.2. Feature Wavelength Extracted by CARS

**Figure 4:**
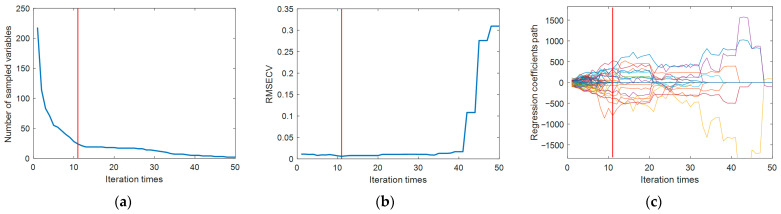
Selection process of CARS variables (different color curves represent different variables) as the number of samples increases. (**a**) Trends in the number of sampled variables. (**b**) Trends in RMSECV values. (**c**) Trends in regression coefficients for each variable.

**Figure 5:**
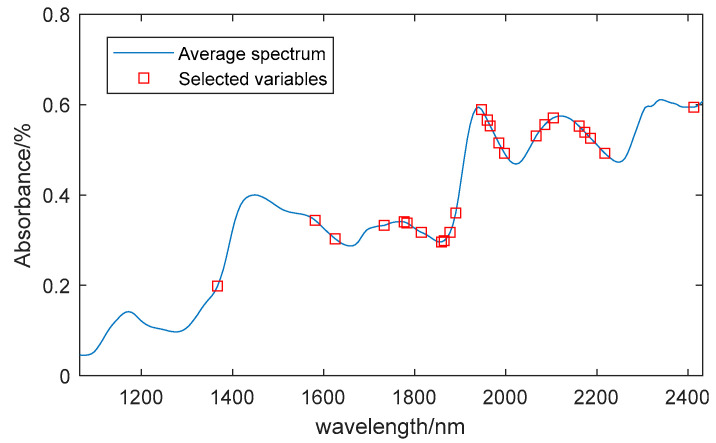
Feature wavelengths extracted by CARS.

We used CARS to extract the characteristic wavelengths of the moisture content, set the number of MC samples to 50, and used a 10-fold cross-validation method. It can be seen from Figure 4a that with the increase in sampling times, the number of variables selected by CARS gradually decreases, and the trend of this change is from a rapid decrease to a more gradual approach to stability. Figure 4b shows the trend of interactive validation error rate during the selection process, with the lowest error rate observed when the sampling time is 11. Figure 4c shows the change in the regression coefficient path as the number of samples increases. When the number of samples is 11, the RMSECV is minimized. Through CARS selection, 24 feature wavelengths were identified, including 1367 nm, 1581 nm, 1625 nm, 1733 nm, 1777 nm, 1783 nm, 1814 nm, 1859 nm, 1865 nm, 1877 nm, 1890 nm, 1947 nm, 1959 nm, 1966 nm, 1985 nm, 1997 nm, 2066 nm, 2085 nm, 2104 nm, 2161 nm, 2174 nm, 2186 nm, 2218 nm, and 2413 nm, accounting for 11% of the total wavelengths. Figure 5 shows the locations of these feature wavelengths in the spectrum.

#### 3.4.3. Feature Wavelength Extracted by UVE

**Figure 6:**
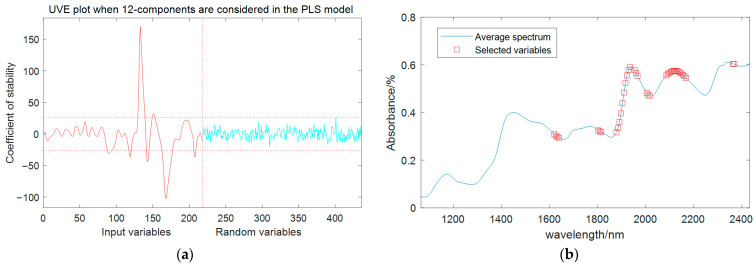
UVE feature extraction results of moisture content. (**a**) Stability distribution curve of UVE-PLS model. (**b**) Locations of selected variables.

When the potential variable was set to 12, the PLS model had the minimum RMSECV value of 0.3036. As shown in Figure 6a, there are 218 wavelength variables on both sides of the vertical dashed line, with the left side being the spectral variable matrix of maize seeds and the right side being the added random noise matrix with the same number of spectral variables. The two horizontal dashed lines represent the thresholds for variable selection, which are determined by the stability of the random variable. The corresponding variables outside the dashed lines are the selected characteristic wavelengths. Through UVE selection, 39 feature wavelengths were identified, including 1619 nm, 1625 nm, 1632 nm, 1638 nm, 1802 nm, 1808 nm, 1814 nm, 1877 nm, 1884 nm, 1890 nm, 1896 nm, 1903 nm, 1909 nm, 1915 nm, 1922 nm, 1928 nm, 1934 nm, 1953 nm, 1959 nm, 1966 nm, 2003 nm, 2010 nm, 2016 nm, 2085 nm, 2092 nm, 2098 nm, 2104 nm, 2111 nm, 2117 nm, 2123 nm, 2129 nm, 2136 nm, 2142 nm, 2148 nm, 2155 nm, 2161 nm, 2167 nm, 2363 nm, and 2369 nm, accounting for 17.9% of the total wavelengths. Figure 6b shows the locations of the characteristic wavelengths in the spectrum.

### 3.5. Establishment of Regression Model

**Figure 7:**
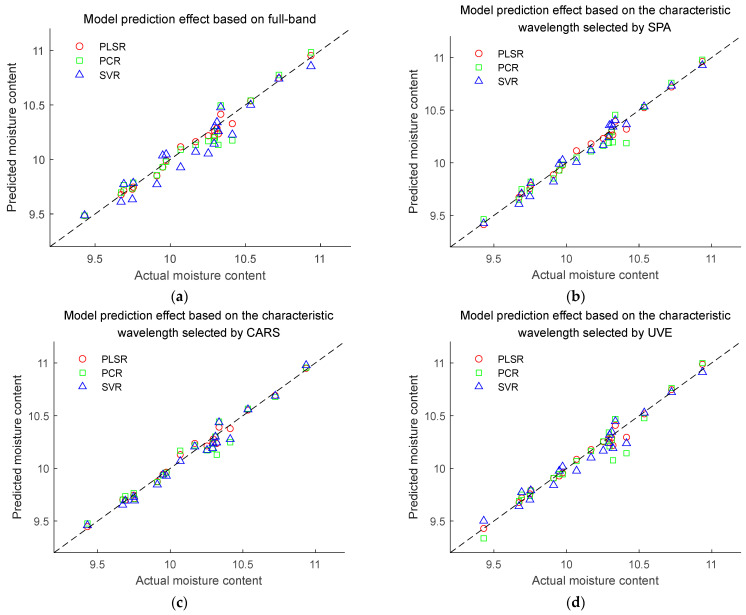
Prediction effect of moisture content models based on PLSR, PCR, and SVR. (**a**) Model prediction effect based on full-band. (**b**) Model prediction effect based on the characteristic wavelength selected by SPA. (**c**) Model prediction effect based on the characteristic wavelength selected by CARS. (**d**) Model prediction effect based on the characteristic wavelength selected by UVE.

Combining seven preprocessing methods and three feature wavelength selection algorithms, we established PLSR regression models and calculated the RMSECV using the leave-one-out cross-validation method as an evaluation metric for the models. We found that normalization was still the optimal preprocessing method. After preprocessing the spectra by normalization algorithm, PLSR, PCR, and SVMR models were established for the full band and characteristic wavelengths, respectively. The root mean square error of prediction (RMSEP) value of the prediction set was used as an indicator to evaluate the prediction performance of the models. The model prediction results are shown in Table 3 and Figure 7.

Among the models established based on the 1100–2498 nm spectral range, the PLSR model exhibits lower RMSEP and RMSECV values, indicating that the PLSR model based on broad-spectrum data exhibits better prediction performance and stability. As shown in Table 3, among the models built with feature wavelengths selected by SPA, CARS, and UVE algorithms, the model based on the SPA algorithm showed a lower RMSEP value compared to the models built with the full bands. However, CARS and UVE algorithms did not significantly improve the model’s predictive performance or even deteriorate it, but they effectively reduced the dimensionality of the spectrum. Among the models built with feature wavelengths, the SPA-PLSR model had the lowest RMSEP value of 0.0257, indicating that SPA selected feature wavelengths for modeling and prediction with good results, likely due to SPA’s effective reduction of spectral collinearity. Therefore, the normalization-SPA-PLSR model was selected as a visual prediction model for maize seed moisture content.

### 3.6. Visualization Analysis of Moisture Content in Maize Seeds

During the harvesting, processing, and storage of corn, it is impossible to directly determine the moisture content using the naked eye. However, using the predictive model, it is possible to calculate the predicted value of the moisture content for each pixel on the hyperspectral image, obtain a grayscale image, and then perform pseudo-color transformation on the grayscale image to obtain a visualization of the moisture content of the maize seeds.

**Figure 8:**
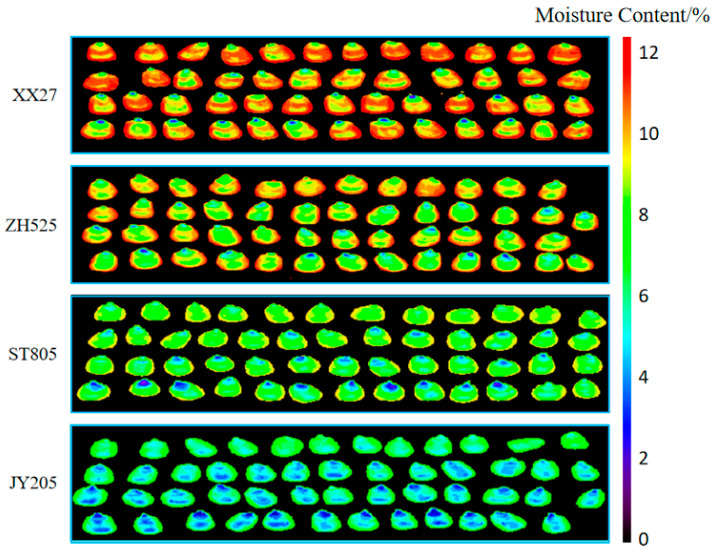
Visualization of corn moisture content.

Figure 8 presents a visualization of the moisture content of four varieties of maize seeds predicted by the normalization-SPA-PLSR model. The color gradient bar represents the moisture content from low to high, ranging from 0 to 12%. The average moisture content of XX27 is 11.53%, ZH525 is 10.16%, ST805 is 8.78%, and JY205 is 7.45%. From Figure 8, it can be seen that the moisture content of different varieties of maize seeds varies in color, and the color differences are significant. Although there are differences in the color of different grains in the same image, the differences are small. Visualizing the hyperspectral images of 20 varieties of maize seeds in the prediction set, the results show that different moisture contents of maize seeds correspond to different colors, and the range of moisture content can be determined by the change in the image color.

## 4. Discussion

In this study, we propose and develop a fast and non-destructive model which is capable of measuring moisture content. The performance of our proposed normalization-SPA-PLSR model is mainly evaluated by $R^{2}$ and RMSE. On the training set, $R_{C}^{2}$ = 0.9917 and RMSEC = 0.0343, indicating that the model can accurately fit the training data. On the test set, $R_{P}^{2}$ = 0.9914 and RMSEP = 0.0257, indicating that the model can make good predictions on unknown data. In addition, the RMSEC is slightly higher than RMSEP, which may be related to the different distributions of sample features in the test set and the training set. Using image processing technology, the moisture content of maize seeds was visualized, and the moisture content range of seeds was visually represented by color. The application and promotion of hyperspectral imaging technology in agriculture provides technical support.

Previous studies lacked in-depth analysis and research on methods for preprocessing spectral data and extracting feature wavelengths. This study addresses this gap and enhances the prediction accuracy of moisture content. Additionally, a method for visualizing moisture content in maize seeds has been introduced. Compared to traditional measurement methods, it offers advantages of being non-destructive, rapid, and accurate, offering technical support for the harvesting, storage, and processing of maize seeds. However, this study also faces shortcomings and areas for improvement. Significant variations exist between the endosperm and embryo surfaces of maize seeds. This study focuses solely on the endosperm surface, complicating the measurement process and potentially introducing measurement errors. In future studies, it might be advisable to consider incorporating methods for identifying the placement of maize seeds and detecting the moisture content on the embryo surface, thereby enhancing the model’s accuracy and applicability.

## 5. Conclusions

This study uses hyperspectral imaging technology to detect the moisture content of maize seeds quickly and non-destructively. The main conclusions are as follows:

1. Using seven preprocessing methods to establish a PLSR model for spectral data in the 1100–2498 nm band, it was found that the normalization method resulted in the highest $R_{C}^{2}$ value, the lowest RMSECV value, and the best model stability.
2. SPA, CARS, and UVE were employed to extract characteristic wavelengths. These methods resulted in the extraction of 17, 24, and 39 wavelengths, respectively, which constitute 7.8%, 11%, and 17.9% of the spectral data, reducing redundancy and irrelevant information, effectively lowering the dimensionality of the spectral data, speeding up data processing, and facilitating the construction of more accurate and robust prediction models.
3. By integrating the feature wavelength extraction method with the modeling approach, we evaluated the efficacy of 12 models. The normalization-SPA-PLSR model exhibited notably high $R_{C}^{2}$ and $R_{P}^{2}$ values of 0.9917 and 0.9914, respectively, along with notably low RMSEP and RMSECV values of 0.0343 and 0.0257, respectively. This model demonstrated commendable stability and predictive accuracy, allowing for rapid, accurate, and loss-free detection of the moisture content in maize seeds.
4. When we visualized the 20 hyperspectral images in the prediction set, the color of the visualized images of maize seeds varied according to moisture content. The moisture content range of the maize seeds can thus be determined by the color changes in the images.

In summary, hyperspectral imaging technology can achieve rapid and non-destructive detection of the moisture content of maize seeds. The established normalization-SPA-PLSR model demonstrates reliable predictive performance, offering a methodological basis for further research on maize seed quality detection and system development.

## Figures and Tables

**Table 1:** Moisture content of samples.

Sample Set	Number of Samples	Moisture Content %			
		Maximum Value	Minimum Value	Average Value	Standard Deviation
Calibration set	60	11.9930	7.3770	9.118	0.3786
Validation set	20	11.9770	7.4300	9.2719	0.3900
Total sample	80	11.9930	7.3770	9.2335	0.3804

**Table 2:** PLSR model based on different pretreatment methods.

Pretreatment Method	PCs	Calibration Set		Validation Set	
		$R_{C}^{2}$	RMSEC	$R_{CV}^{2}$	RMSECV
No pretreatment	7	0.9772	0.0571	0.9720	0.0632
Moving Average	7	0.9789	0.0553	0.9746	0.0589
S–G smoothing	7	0.9792	0.0549	0.9732	0.0596
Normalization	7	0.9890	0.0378	0.9886	0.0375
Baseline	7	0.9835	0.0485	0.9791	0.0548
SNV	9	0.9842	0.0526	0.9811	0.0497
MSC	7	0.9774	0.0568	0.9723	0.0631
Detrending	8	0.9883	0.0406	0.9730	0.0624

**Table 3:** Performance of models based on different characteristic wavelength selecting methods.

Model	Bands	PCs	Calibration Set		Validation Set		Prediction Set	
			$R_{C}^{2}$	RMSEC	$R_{CV}^{2}$	RMSECV	$R_{P}^{2}$	RMSEP
PLSR	218	7	0.9878	0.0414	0.9811	0.0525	0.9848	0.0366
PCR	218	7	0.9654	0.0699	0.9545	0.0815	0.9371	0.0687
SVMR	218		0.9436	0.0920	0.8701	0.1379	0.9193	0.0895
SPA-PLSR	17	7	0.9917	0.0343	0.9891	0.0401	0.9914	0.0257
SPA-PCR	17	7	0.9719	0.0630	0.9620	0.0742	0.9547	0.0590
SPA-SVMR	17		0.9853	0.0468	0.9672	0.0691	0.9798	0.0456
CARS-PLSR	24	8	0.9872	0.0426	0.9818	0.0520	0.9889	0.0315
CARS-PCR	24	8	0.9618	0.0735	0.9472	0.0877	0.9550	0.0611
CARS-SVMR	24		0.9747	0.0619	0.9566	0.0817	0.9738	0.0470
UVE-PLSR	39	9	0.9899	0.0378	0.9878	0.0426	0.9854	0.0309
UVE-PCR	39	8	0.9333	0.0971	0.9210	0.1071	0.9322	0.0617
UVE-SVMR	39		0.9714	0.0695	0.9634	0.0844	0.9598	0.0605

## Data Availability

All relevant data presented in the article are stored according to institutional requirements and, as such, are not available online. However, all data used in this manuscript can be made available upon request to the authors.
